# Supplementary material for: Type I toxin-antitoxin systems contribute to the maintenance of mobile genetic elements in Clostridioides difficile
Source: Commun Biol. 2020 Nov 27;3:718. doi: 10.1038/s42003-020-01448-5 (PMC7699646; doi:10.1038/s42003-020-01448-5)
Supplement: Supplementary file 1 — Supplementary Information [file 42003_2020_1448_MOESM1_ESM.pdf]

Type I toxin-antitoxin systems contribute to the maintenance of mobile genetic elements in *Clostridioides difficile*

Johann Peltier, Audrey Hamiot, Julian R. Garneau, Pierre Boudry, Anna Maikova, Eliane Hajnsdorf, Louis-Charles Fortier, Bruno Dupuy and Olga Soutourina

Supplementary information

Supplementary Figures, Supplementary Table Supplementary methods and Supplementary references

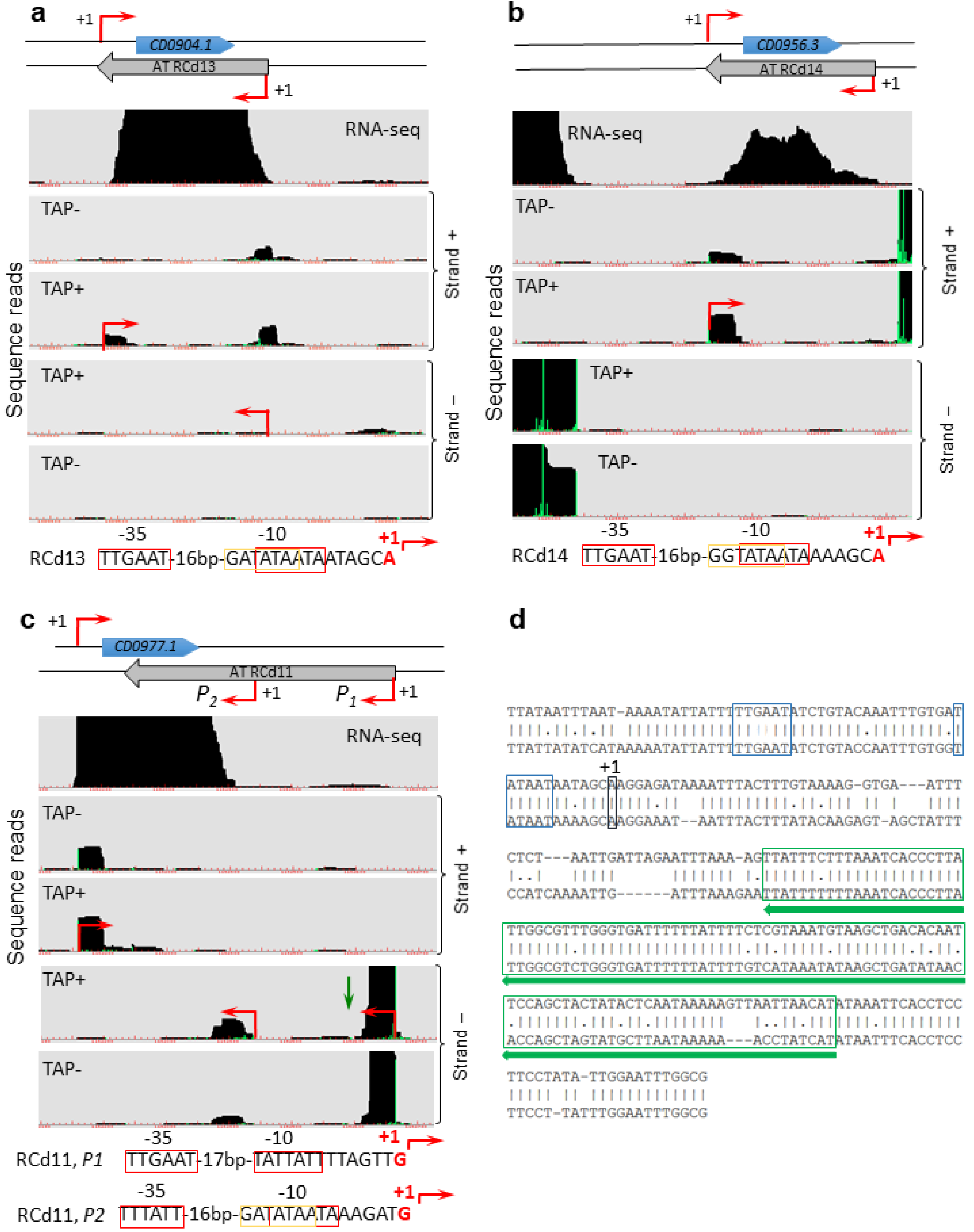

Supplementary Figure 1. RNA-seq and TSS mapping profiles for the TA loci in *C. difficile* strain 630. The TAP-/TAP+ profile comparison for 5'-end RNA-seq data is aligned with RNA-seq data for *CD0904.1*-RCd13 (**a**) and *CD0956.3*-RCd14 (**b**) and *CD0977.1*-RCd11 (**c**) TA genomic regions. The TSS are indicated by red broken arrows in accordance with the positions of 5'-transcript ends shown by vertical green lines on the sequence read graphs corresponding to TSS. Potential processing site is shown in (**c**) by vertical green arrow. 5'-end sequencing data show 51-bp reads matching to the 5'-transcript ends, while RNA-seq data show reads covering the whole transcript. Coding sequences are indicated by blue arrows and the regulatory RNAs are indicated by grey arrows. The promoter regions of the antitoxins are shown. The promoter region of RCd14 antitoxin was deduced from the alignment in (**d**). **d** Alignment between the sequences of *CD0904.1*-RCd13 (top line) and *CD0956.3*-RCd14 (bottom line) using EMBOSS Needle. Green boxes show the open reading frames of *CD0904.1* and *CD0956.3* and green arrows indicate the direction of the genes. Blue boxes show the -10 and the -35 boxes of the promoter regions and black boxes show the transcription start site of RCd13 and RCd14. In **a**, **b** and **c**, the red boxes show the Sigma A-dependent promoter -10 and -35 elements and the yellow boxes show the Sigma B-dependent promoter element.

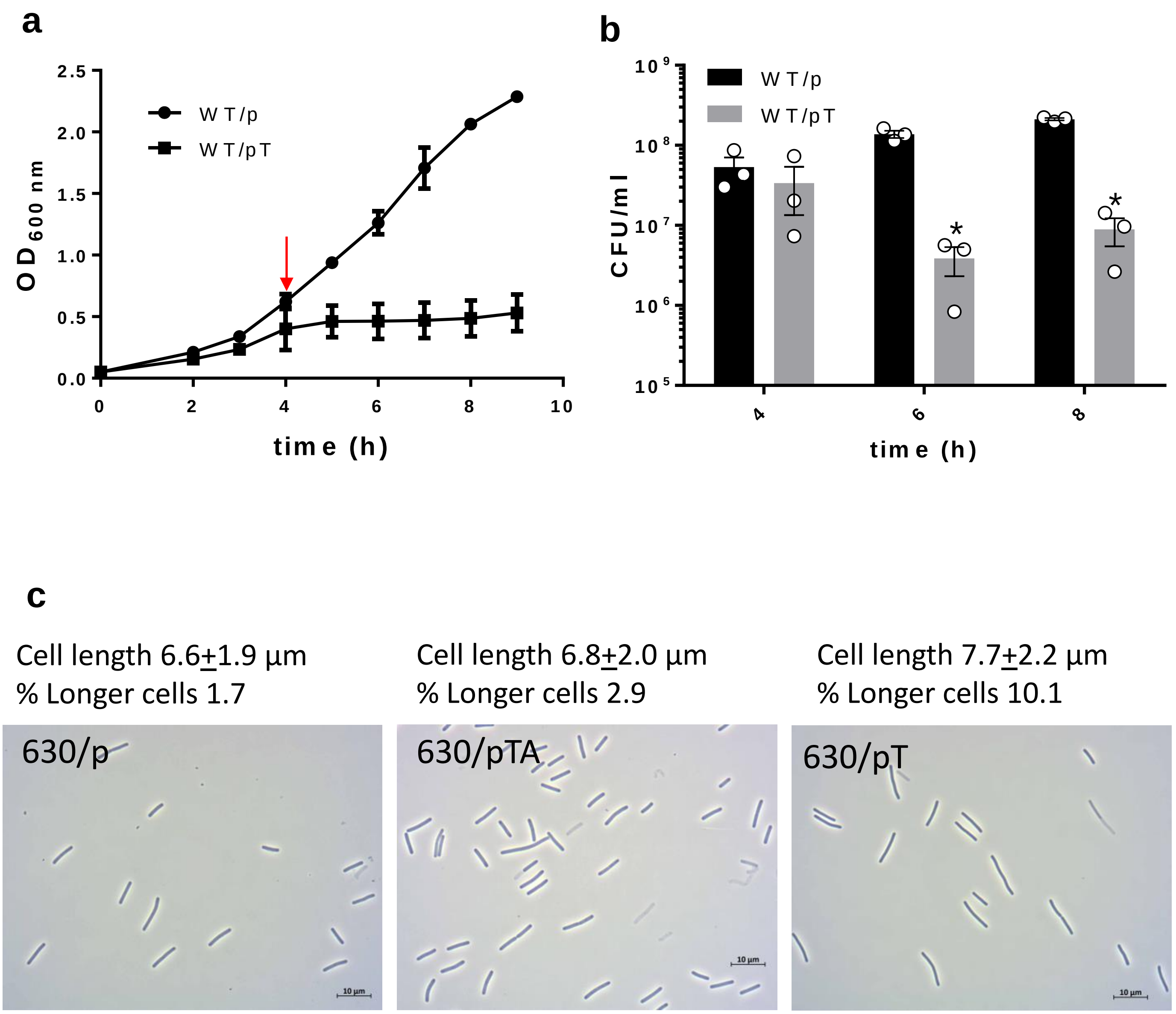

Supplementary Figure 2. Impact of toxin CD0977.1 expression on cell viability and morphology. Growth (**a**) and viability (**b**) of *C. difficile* 630Δ*erm* strain carrying the pRPF185-based plasmids (empty: p or with *CD0977.1* toxin gene under the control of the P_{tet} promoter: pT) in TY broth in presence of 250 ng/ml ATc. The time point of ATc addition is indicated by a vertical arrow. Values represent means and error bars represent standard error of the means (N = 3 biologically independent samples). * $P \leq 0.05$ by a Student's *t* test. **c** Selected images from light microscopy observations of 630/p, 630/pT and 630/pTA strains grown in TY broth for 1 h at 37°C after the addition of 250 ng/mL ATc. Cell length was estimated using the ImageJ software for at least 115 cells per strain. The mean values with standard deviation are indicated for each strain, as well as the proportion of cells with length above 2 standard deviations relative to the 630/p control strain mean length. Error bars represent standard deviations.

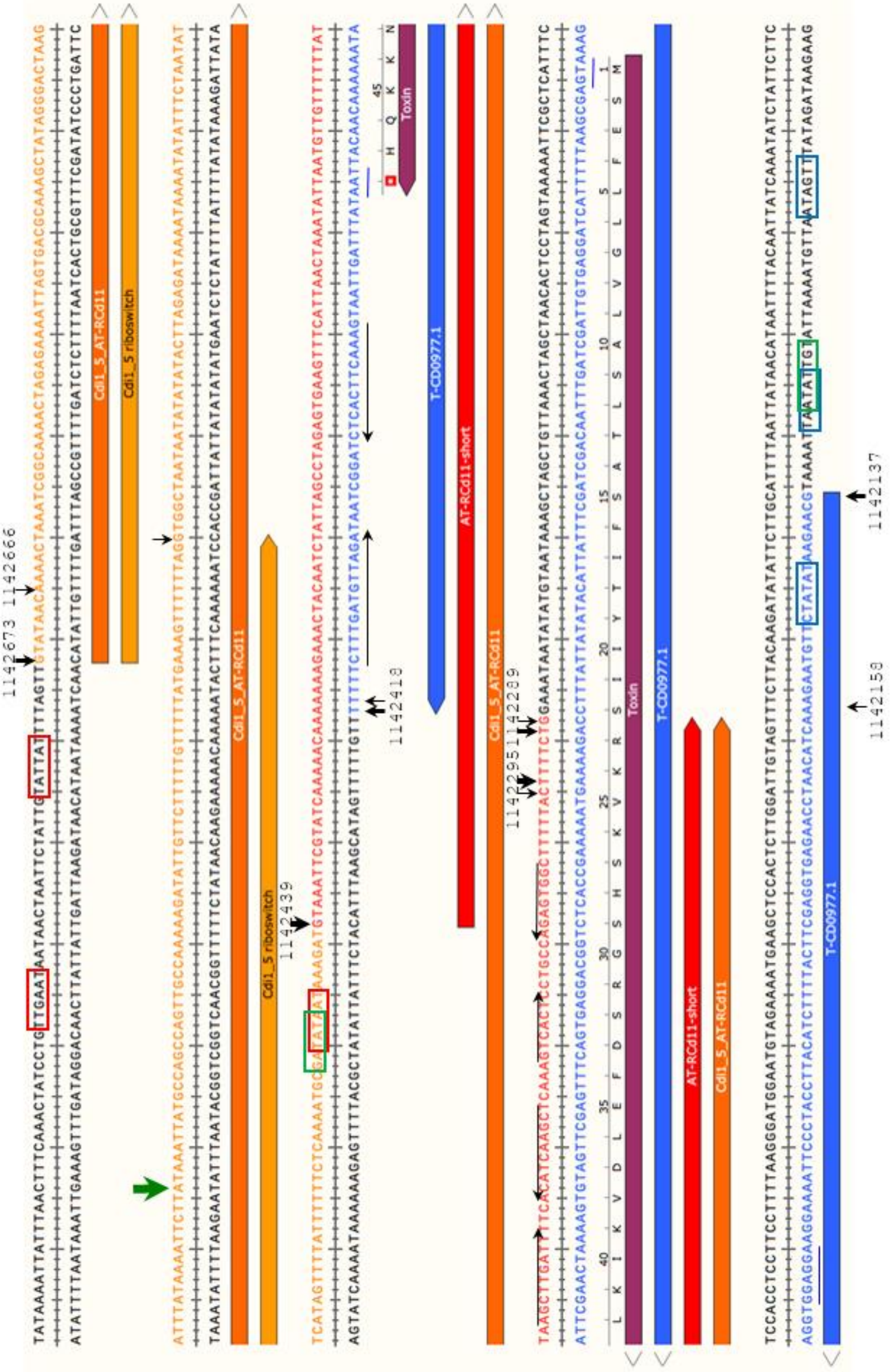

Supplementary Figure 3. Sequence of type I *CD0977.1*-RCd11 TA locus in *C. difficile*. The thick horizontal arrows below the double-stranded sequences show the toxin and antitoxin transcripts and the direction of transcription. The transcriptional start sites for sense and antisense transcripts identified by 5’/3’RACE and TSS mapping are indicated by vertical arrows with their genomic location. Line thickness corresponds to the proportion of observed extremities. The genomic location of 5’- and 3’-ends of the transcripts are indicated above the sequence. Potential processing site is shown by vertical green arrow. The inverted repeats at the position of transcriptional terminators are indicated by thin black arrows. The positions of Sigma A-dependent -10 and -35 promoter elements of antitoxin (AT) are shown in red boxes. The positions of Sigma A-dependent -10 and -35 elements promoter, ribosome binding site, translation initiation codon and stop codon of toxin (T) mRNA are shown in blue boxes. The positions of Sigma B-dependent promoter elements are shown in green boxes for both TA genes.

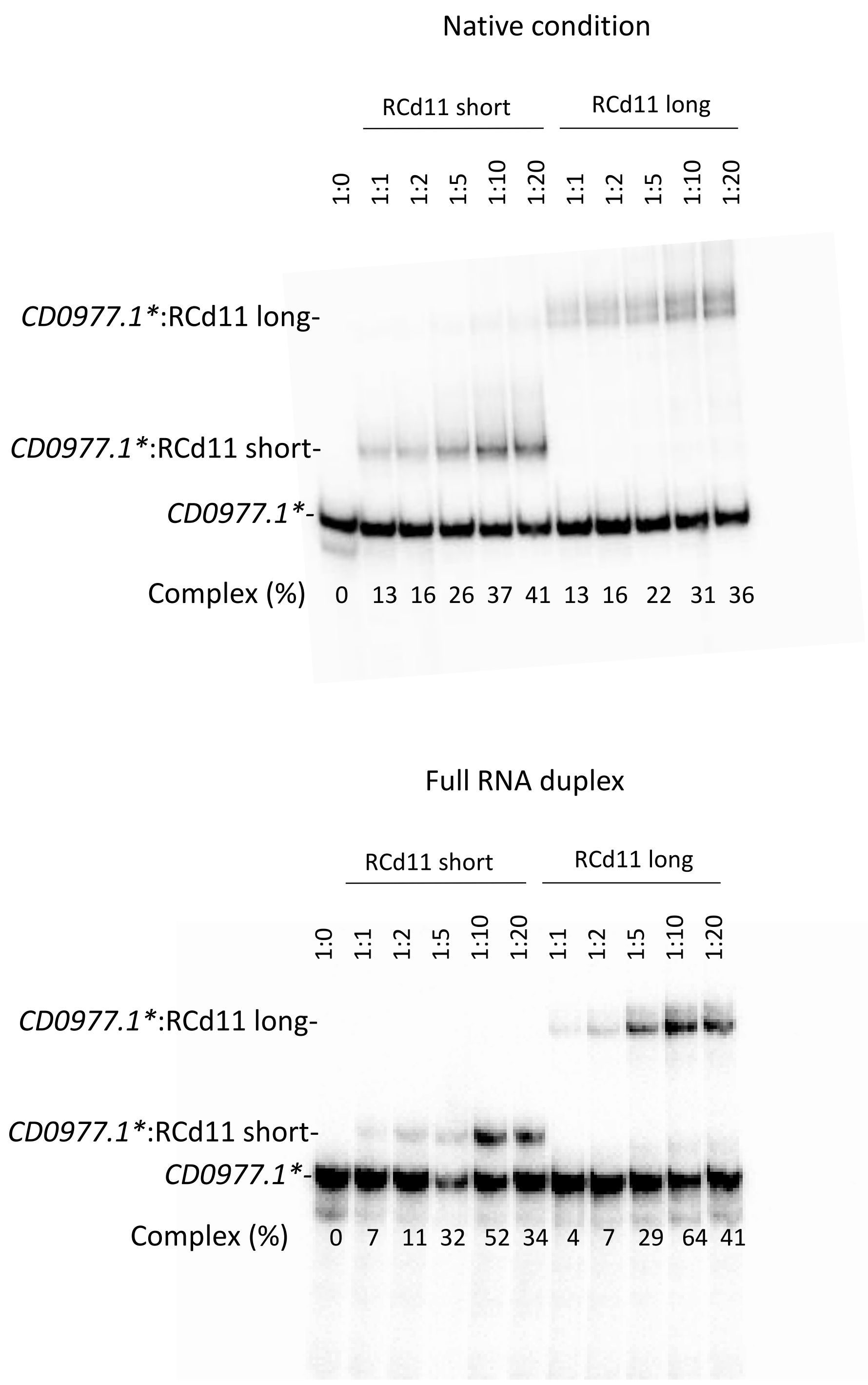

Supplementary Figure 4. Analysis of TA RNA duplex formation by RNA band shift assay. Radiolabeled *CD0977.1* transcript was incubated with increasing concentrations of RCd11 short or long RNAs under two different conditions referred as native and full RNA duplex conditions. Native *CD0977.1*:RCd11 complexes were formed at 37 °C for 5 min in TMN buffer, and full duplexes were obtained after a denaturation-annealing treatment in TE Buffer (2 min 90°C, 30 min 37°C). The complexes were immediately loaded on native polyacrylamide gels to control for hybridization efficiency. RNA levels were quantified by phosphoimagery and complex proportion is indicated.

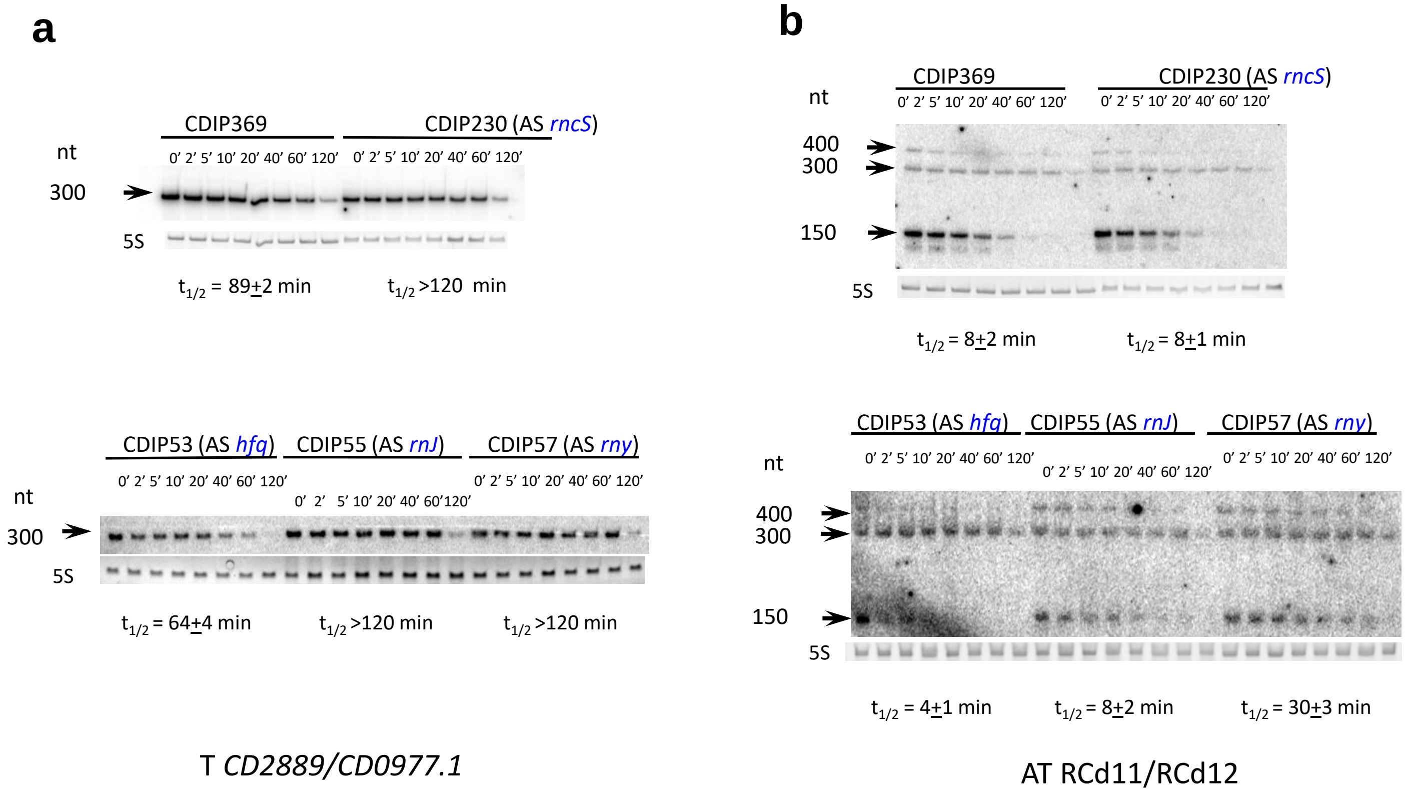

Supplementary Figure 5. Northern blots showing the stability of *CD0977.1/CD2889* toxin and RCd11/RCd12 antitoxin transcripts in strains depleted for RNase III, RNase Y, RNase J and Hfq. a panel for *CD0977.1/CD2889* toxin and **b** panel for RCd11/RCd12 antitoxin. To determine half-lives, samples were taken at the indicated time points after the addition of 200 µg/mL rifampicin. RNAs were extracted from strains CDIP369 (630/p), CDIP230 expressing an antisense RNA for the *rncS* gene encoding RNase III (AS *rncS*), CDIP53 strain expressing an antisense RNA for the *hfq* gene (AS *hfq*), CDIP55 strain expressing an antisense RNA for the *rnJ* gene encoding RNase J (AS *rnJ*) and CDIP57 strain expressing an antisense RNA for the *rny* gene encoding RNase Y (AS *rny*). All Northern blots were probed with a radiolabelled oligonucleotide specific to the toxin (T CD0977.1/CD2889) or the antitoxin (AT RCd11/RCd12) transcript and 5S RNA at the bottom serves as loading control. The relative intensities of the bands were quantified using ImageJ software. The half-lives for toxin and antitoxin transcripts have been estimated from three independent experiments.

Fig. 2b

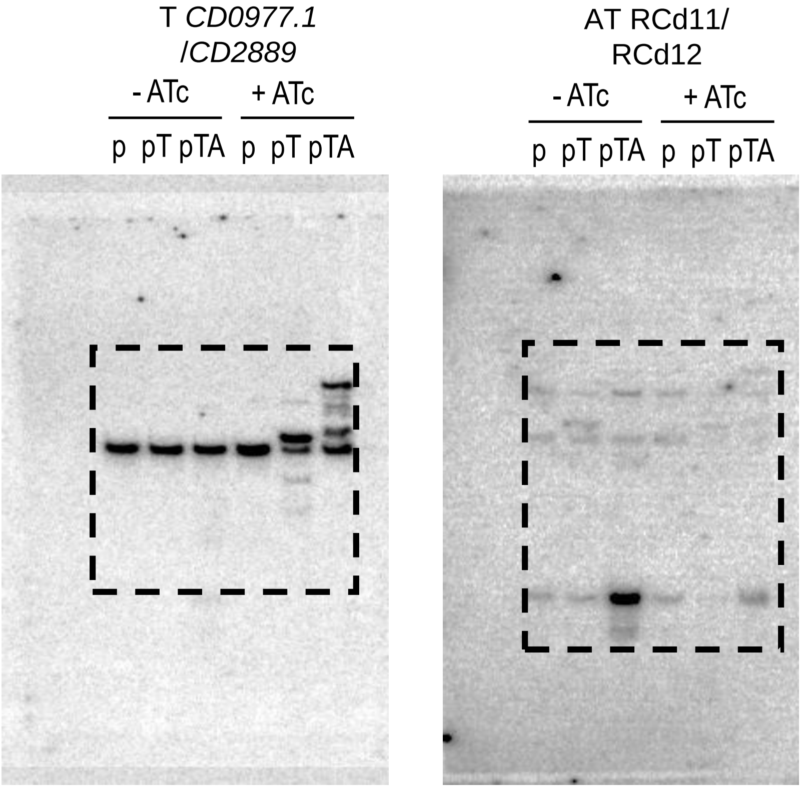

Fig. 2c

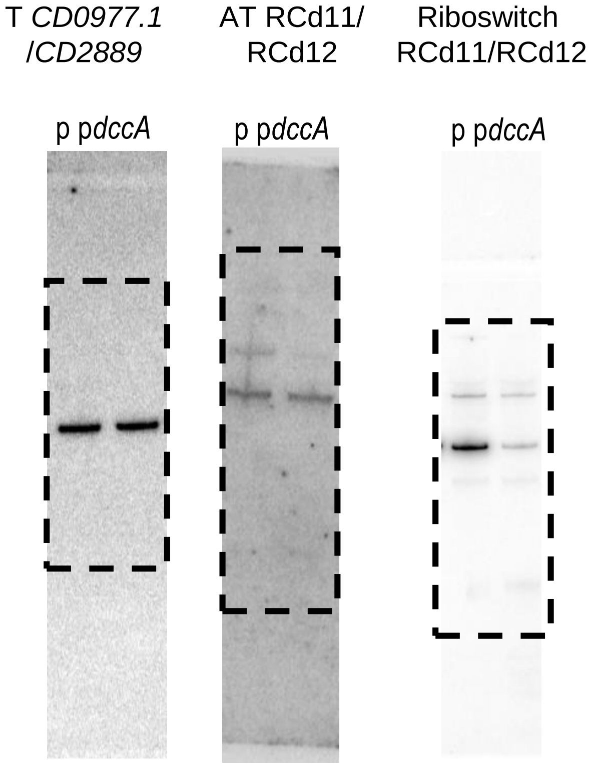

Fig. 2d

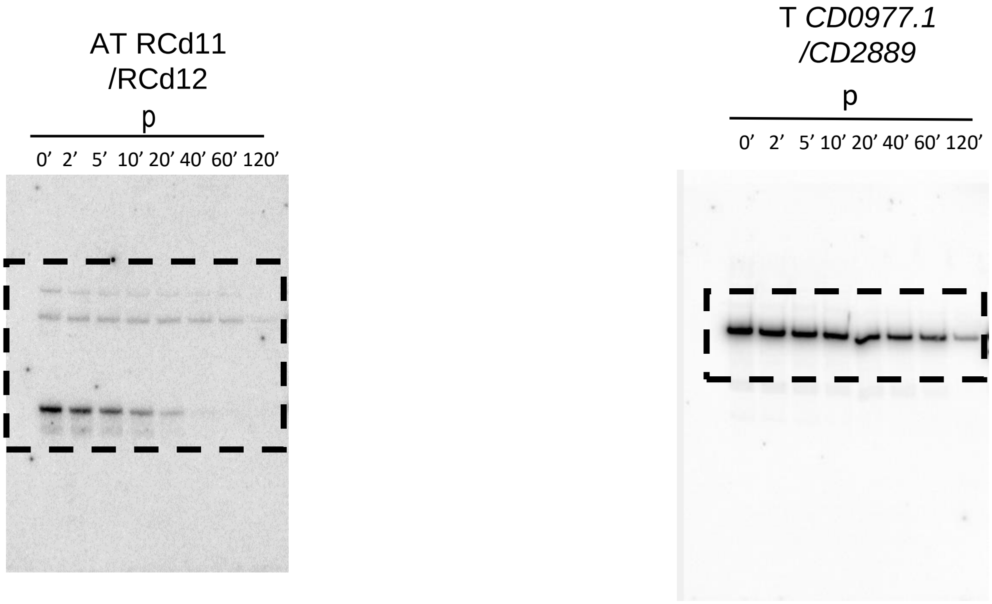

Supplementary Figure 6. Uncropped Northern-blots images corresponding to Figure 2b, 2c and 2d

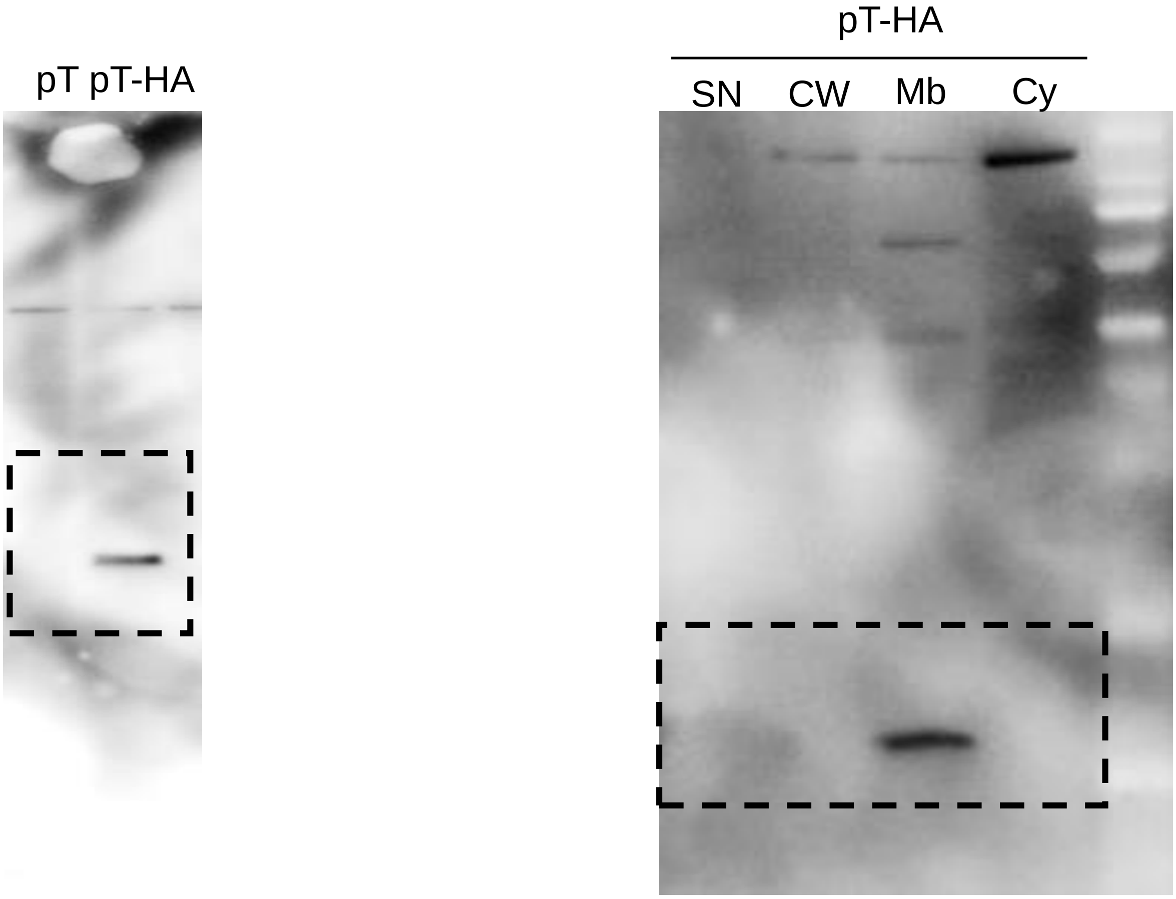

Supplementary Figure 7. Uncropped Western-blots images corresponding to Figure 2e.

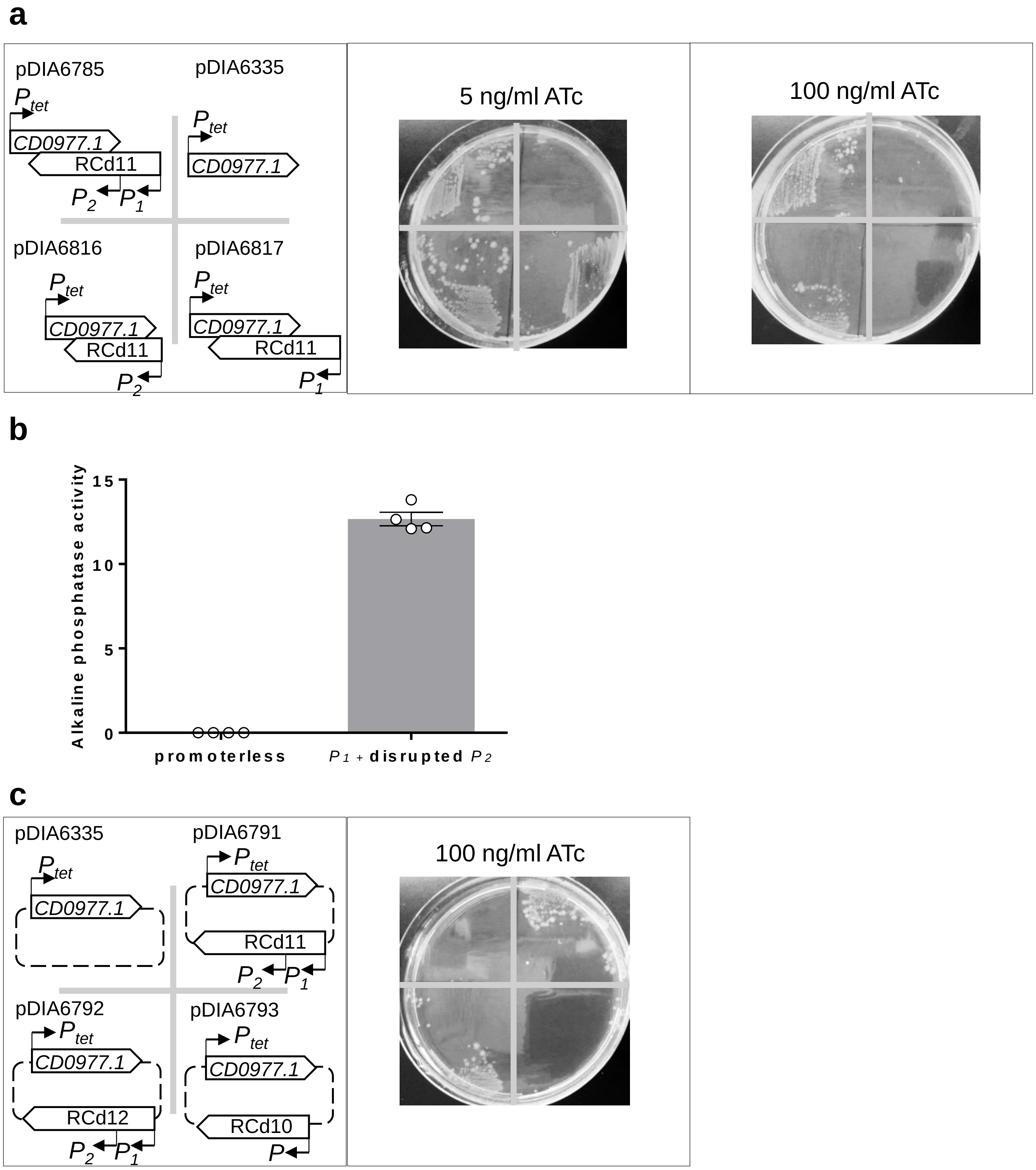

Supplementary Figure 8. Impact of toxin-antitoxin co-expression on growth. The effect on the toxicity of *CD0977.1* of long and short antitoxin transcripts expressed *in cis* (**a**) and *in trans* (**c**) was assessed. **a** and **c** Growth of *C. difficile* 630Δ*erm* strains harbouring the pRPF185-based plasmids on BHI agar plates supplemented with Tm and the indicated concentration of ATc inducer after 24 hrs of incubation at 37°C. Schematic representations of the constructs are shown. **b** Alkaline phosphatase activity of the RCd11 promoter P_1 + disrupted P_2::*phoZ* reporter fusions and the promoterless *phoZ* measured after 4 h (exponential) of growth in TY broth. Values represent means and error bars represent standard error of the means (N = 4 biologically independent samples). Error bars represent standard error of the means.

a

```
                10        20        30        40        50        60
RCd11   GTAAATTCGTATCAAAAACAAAAAAAGAAACTACAATCTATTAGCCTAGAGTGAAGTTTC
        ::::::::::::::::::::::::::::::::::::::::::::::::::::::::::::
RCd12   GTAAATTCGTATCAAAAACAAAAAAAGAAACTACAATCTATTAGCCTAGAGTGAAGTTTC
                10        20        30        40        50        60

                70        80        90       100       110       120
RCd11   ATTAACTAAATATTAATGTTGTTTTTTATTAAGCTTGATTTTCACATCAAGCTCAAAGTC
        ::::::::::::::::::: :::::::::::: ::::::::: :::::::::::::::::
RCd12   ATTAACTAAATATTAATGTCGTTTTTTATTAAACTTGATTTTTACATCAAGCTCAAAGTC
                70        80        90       100       110       120

               130       140       150
RCd11   ACTCCTGCCAGAGTGGCTTTTTACTTTTCTG
        :::::::::::::::::::::::::::::::
RCd12   ACTCCTGCCAGAGTGGCTTTTTACTTTTCTG
               130       140       150
```

b

```
               30        40          50          60        70
RCd11   AAAAAGAAACTACAATCTATTAGCC--TAGAGTGAAGTT--TCATTAACTAAATATTAAT
        :: ::::: ::::::::::::   :  ::::::::::::  : ::::: : ::: :  ::
RCd10   AAGAAGAA-CTACAATCTATTTTGCGGTAGAGTGAAGTTCATAATTAAATGAATCT--AT
                 10        20        30        40        50

         80        90       100       110       120             130
RCd11   GTTGTTTTTTATTAAGCTTGATTTTCACATCAAGCTCAAAGTCACT------CCTGCCA-
         :::   :: ::  : ::::::::: : :::::  ::  :: ::::      : ::::: 
RCd10   -TTGAACTT-ATGGAACTTGATTTTAAAATCAAATTCCCAGCCACTTTTACTCTTGCCAC
          60         70        80        90      100       110

                    140
RCd11   -----GAGTGGCTTTTTACTTT
             :::::::::::::::::
RCd10   GAGTTGAGTGGCTTTTTACTTT
          120       130
```

Supplementary Figure 9. Nucleotide alignment of antitoxins. a Nucleotide alignment of RCd11 and RCd12 using LALIGN. **b** Nucleotide alignment of RCd11 and RCd10 using LALIGN.

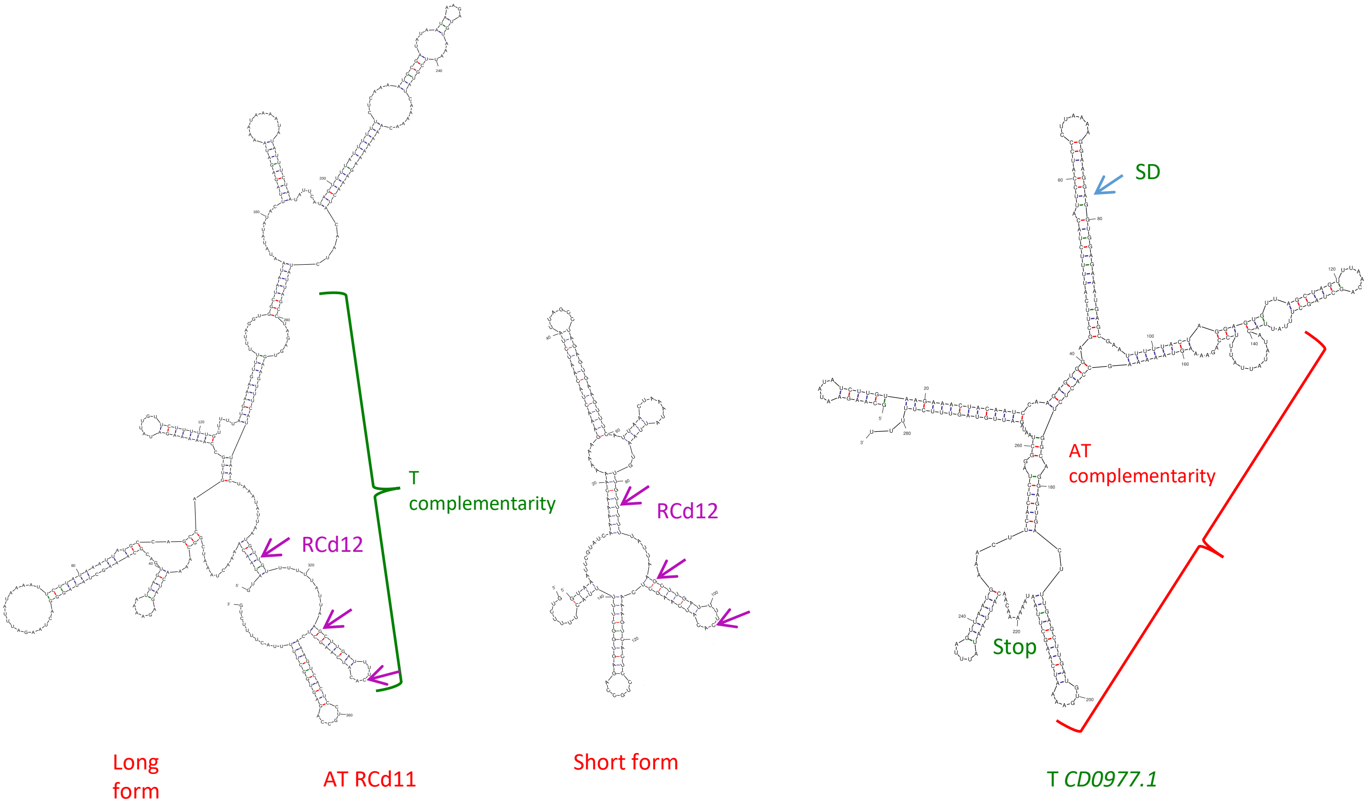

Supplementary Figure 10. Secondary structure prediction of *CD0977.1* mRNAs and corresponding antitoxin RCd11 RNAs. The RNA secondary structure predictions were performed by Mfold software. The predictions for long and short forms of RCd11 antitoxins are shown. Ribosome binding site (SD), translation initiation codon and stop codon positions are highlighted. The positions of mismatches in RCd12 AT sequence are indicated.

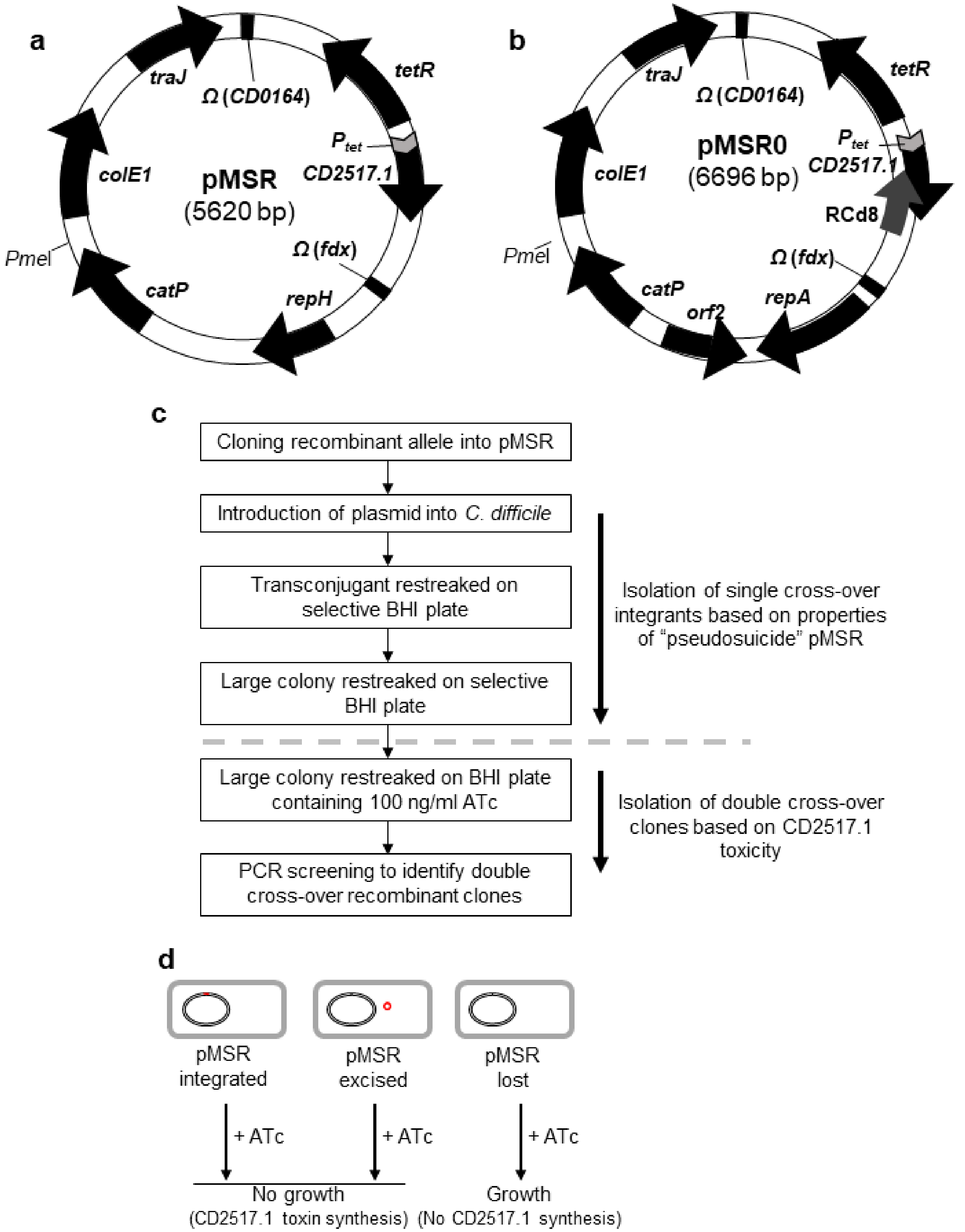

Supplementary Figure 11. Improved vector for efficient gene editing in *C. difficile*. Features of the pMSR (**a**) and pMSR0 (**b**) vectors used for allele exchange in *C. difficile* 630Δ*erm* and *C. difficile* 027 ribotype strains, respectively. The toxin gene *CD2517.1* is under the control of the ATc inducible promoter P_{tet} and the RCd8 antitoxin present in pMSR0 is under control of its own promoter. Schematic overview of the allele exchange protocol (**c**) and of the inducible counterselection method used to isolate double cross-over clones (**d**). Isolated single cross-over integrants were restreaked on ATc-containing agar plates to induce synthesis of toxin CD2517.1. Cells that kept the pMSR plasmid (either integrated or excised) produced CD2517.1 and were selectively killed.

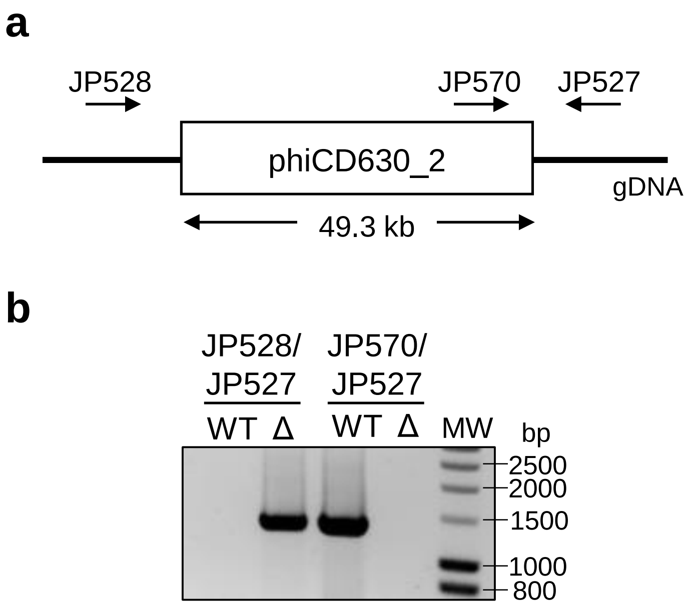

Supplementary Figure 12. Deletion of the phiCD630-2 prophage from *C. difficile* 630Δ*erm* using the newly developed allele exchange method. a Schematic representation of phiCD630-2 in *C. difficile* 630Δ*erm*. The location of primers used to screen for mutants is represented. **b** PCR products amplified using the indicated primers from the parental strain 630Δ*erm* (WT) and the ΔphiCD630-2 strain (Δ). A product of 1,348 bp could be amplified with primers JP528-JP527 if phiCD630-2 had been deleted, whereas a product of 1,339 bp could be amplified with primers JP570-JP527 if phiCD630-2 was still present.

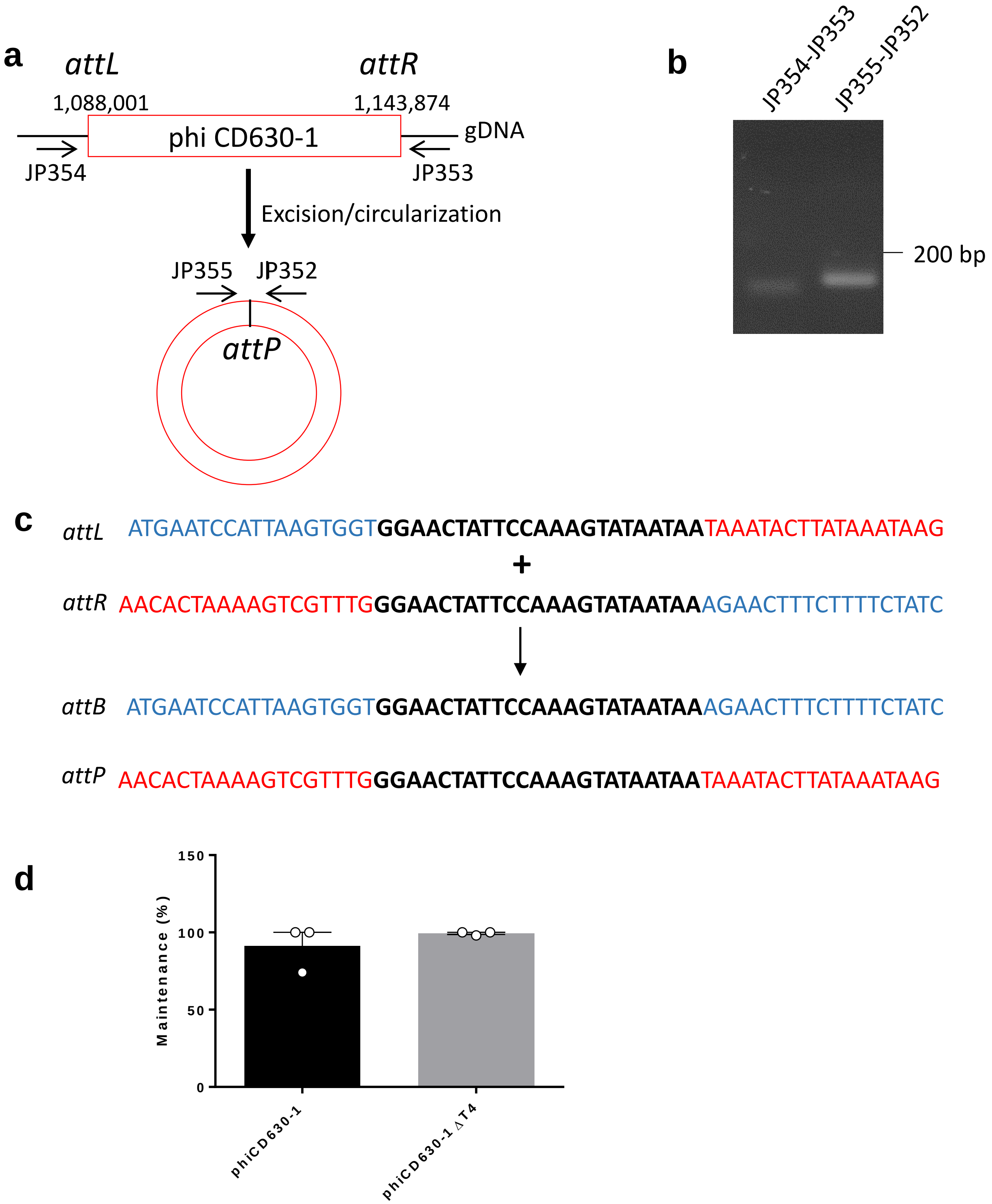

Supplementary Figure 13. Maintenance and site-specific excision of phiCD630-1 from genomic DNA of *C. difficile* 630. a Schematic representation of phiCD630-1 DNA excision from genomic DNA of *C. difficile* 630, and circularization. The location of primers used to demonstrate prophage excision is represented. **b** PCR products amplified using the indicated primers from *C. difficile* 630 genomic DNA. **c** DNA sequences within *attP*, *attB*, *attL* and *attR* as determined by Sanger sequencing. The central identical sequences where recombination occurs are shown in bold. Short segments of sequence surrounding the central identity region are shown in blue (bacterial sequences) and in red (phage sequences). **d** The maintenance of prophage in strains ΔphiCD630-2 phiCD630-1*::erm* and ΔphiCD630-2 phiCD630-1-ΔT4*::erm* was determined after four passages in TY broth by plating serial dilutions on agar plates supplemented or not with 2.5 μg/ml Erm. Values represent means and error bars represent standard error of the means (*N* = 3 biologically independent samples).

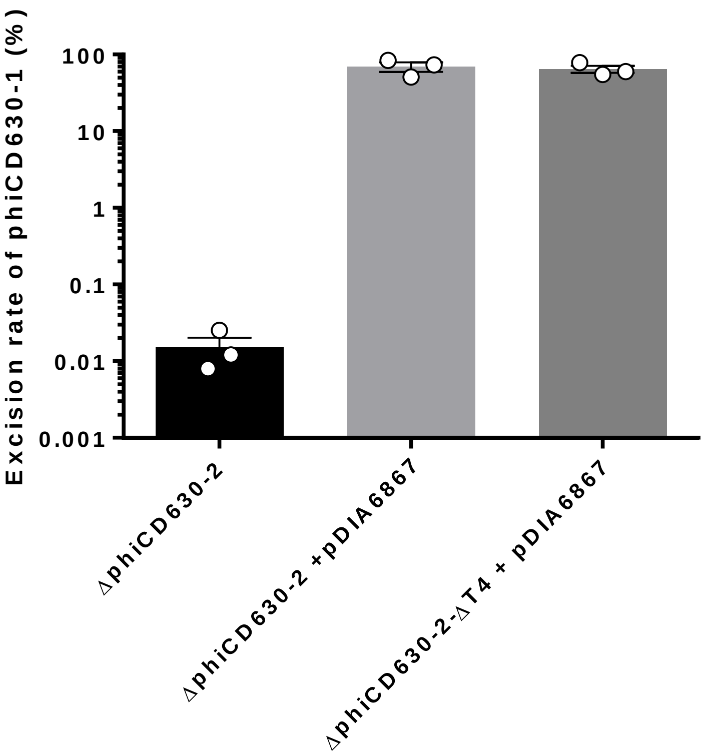

Supplementary Figure 14. Impact of excisionase (CD0912) overproduction on excision of phiCD630-1 and phiCD630-1-ΔT4. The frequency of prophage excision was estimated by quantitative PCR as described in Materials and Methods section. Excision rate of phiCD630-1 was higher in *C. difficile* carrying pDIA6867 (inducible expression of *CD0912*) in presence of 10 ng/ml of the inducer ATc when compared to the ΔphiCD630-2 strain without plasmid and was not impacted by the deletion of toxin genes. Values represent means and error bars represent standard error of the means (N = 3 biologically independent samples).

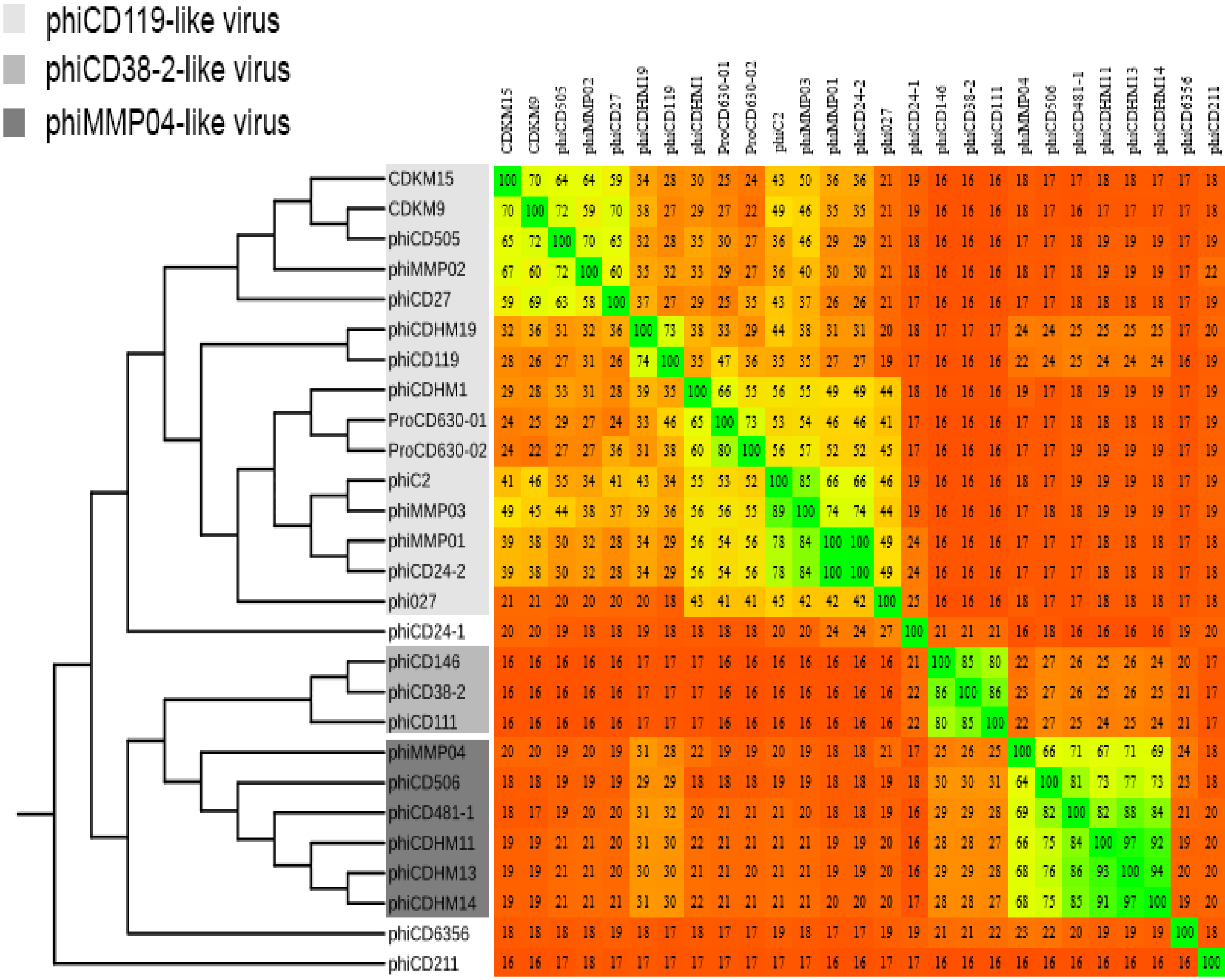

Supplementary Figure 15. Heatmap and phylogenetic tree showing *C. difficile* relatedness for 27 sequenced *C. difficile* phage genomes available in the NCBI database. The Gegenees software (v2.2.1) was used to produce the heatmap of genome similarity. Similarity scores are based on a fragmented all-against-all pairwise alignment using BLASTn and the accurate alignment option (fragment size, 200; step size, 100). The colours reflect the similarity, ranging from low (red) to high (green). Phages were assigned to a genus if they clustered closely to another phage previously described as a member of that genus. The phylogenetic tree is based on the sequence similarity scores from the same whole-genome comparison and was constructed using the neighbour joining method with the SplitsTree4 software (v 4.13.1).

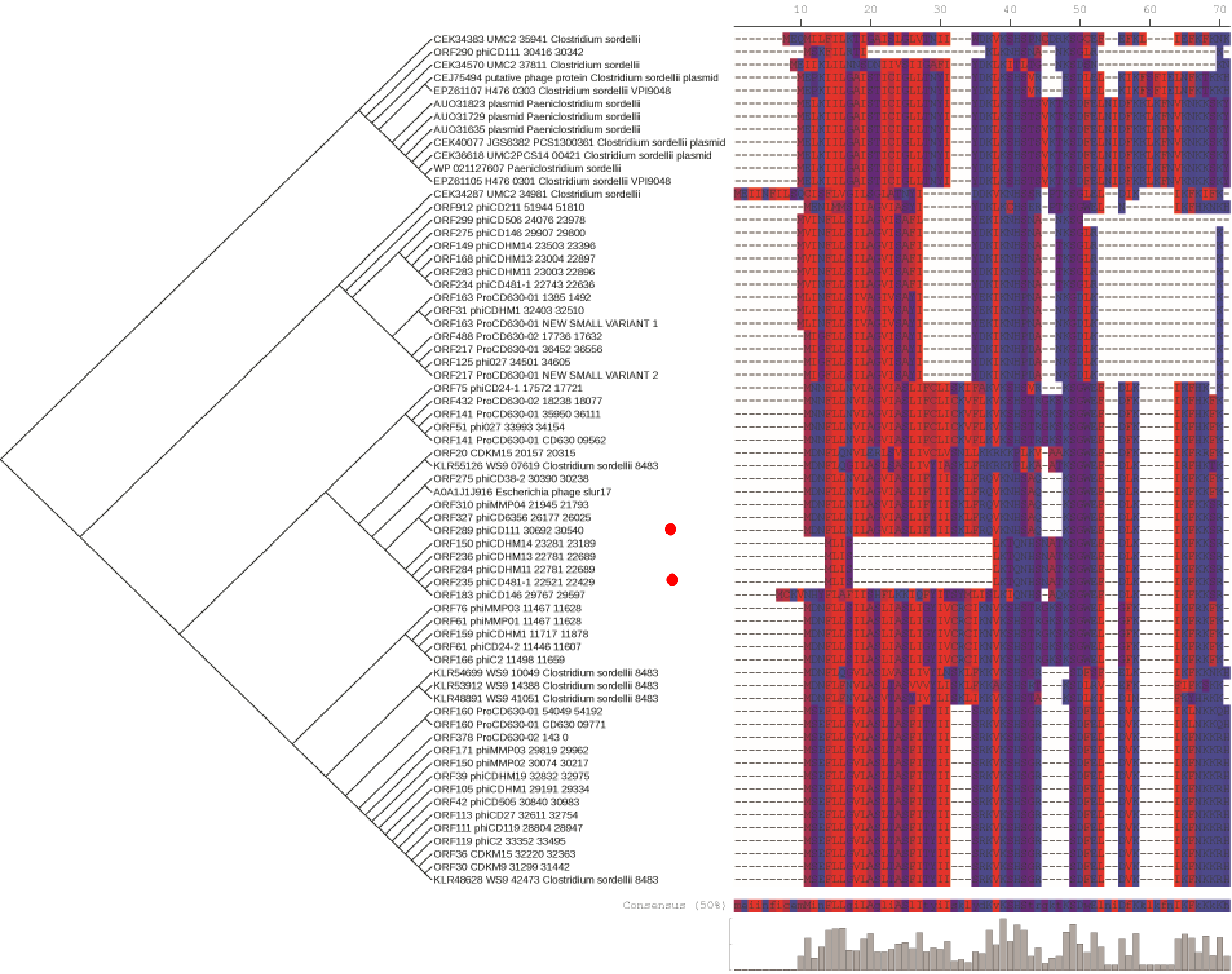

Supplementary Figure 16. Identification of toxin homologs outside *C. difficile* phages. Toxin homologs found outside *C. difficile* phages were aligned. The protein sequence consensus is shown at the bottom. Phylogenic analysis of toxins is also represented. Red dot with tag "small variant 1" indicates CD0904.1. Red dot with tag "small variant 2" indicates CD0956.3.

Name	Description	5'-end RACE position	5'-end TSS mapping position	Strand	3'-end RACE position	Size, nt
**RCd11 (CD630_n00390)**	Antitoxin of TA associated with cdi1_5	1142666, 1142673 1142439	1142666, 1142673 1142439	-	1142531, 1142295, 1142289	143, 151, 385
**CD0977.1**	Toxin of TA associated with cdi1_5	1142137	1142158	+	1142418	282
**RCd12 (CD630_n00980)**	Antitoxin of TA associated with cdi1_4	3379972, 3377979, 3380206	3379972, 3377979, 3380206	+	3380346, 3380352, 3380114	143, 151, 385
**CD2889**	Toxin of TA associated with cdi1_4	3380505	3380505	-	3380223	282

Supplementary Table 1. The antisense RNA and associated toxin mRNA extremity identification for RCd11/RCd12-*CD0977.1/CD2889* by 5'/3'RACE.

The positions of 5'-start and 3'-end of these RNAs were identified by 5'/3'RACE analysis and compared with 5' –end identified by 5'-end RNA-seq analysis (TSS mapping).

Supplementary Methods

For expression of TA genes under the control of their native promoters, the *CD0977.1*-RCd11, *CD0956.2*-RCd10, *CD0904.1*-RCd13, *CD0956.3*-RCd14 TA modules and their associated promoters were amplified by PCR and cloned into BamHI and XhoI sites of pMTL84121 [1].

For inducible expression of *C. difficile* genes, we used the pDIA6103 derivative of pRPF185 vector expression system lacking the *gusA* gene [2]. To assess the activity of the putative toxin CD0977.1 and the functionality of its cognate antitoxin, the *CD0977.1* gene (-66 to +222 relative to the translational start site) and the RCd11-*CD0977.1* region (-220 to +608 relative to the translational start site) were amplified by PCR and cloned into StuI and BamHI sites of pDIA6103 vector under the control of the ATc-inducible P_{tet} promoter; yielding pDIA6335 and pDIA6337.

All other constructs described below were performed with NEBuilder HiFi DNA Assembly (NEB). For this purpose, the pDIA6103 vector was linearized by inverse PCR at the MCS for cloning of genes under the control of the ATc-inducible P_{tet} promoter. To assess the toxicity of CD0904.1 and CD0956.3 and the functionality of their cognate antitoxin, the *CD0904.1* (-43 to + 109 relative to the translational start site) and *CD0956.3* genes (-35 to + 108 relative to the translational start site) as well as the RCd13-*CD0904.1* (-43 to +230 relative to the translational start site) and the RCd14-*CD0956.3* regions (-35 to + 248 relative to the translational start site) were amplified by PCR and cloned into the linearized pDIA6103 vector, yielding pDIA6866, pDIA7030, pDIA6934 and pDIA7031, respectively. For inducible toxin expression and co-expression of its cognate antitoxin, the RCd11-*CD0977.1* TA region with the two RCd11 promoters (-66 to +608 relative to the translational start site of *CD0977.1*) or the RCd11-*CD0977.1* TA region with the P_2 promoter only (-66 to +306 relative to the translational start site of *CD0977.1*) were amplified by PCR and cloned

into the linearized pDIA6103 vector, yielding pDIA6785 and pDIA6816, respectively. For inducible toxin expression and co-expression of its cognate antitoxin with the promoter P_1 only, the identified transcriptional start site (TSS) G of P_2 promoter was replaced with a T and the -10 box TATAAT was replaced with TTTTTT, using an inverse PCR approach, to disrupt the P_2 promoter; yielding pDIA6817. To investigate the action of cognate (RCd11) and non-cognate (RCd10 and RCd12) antitoxins on toxin CD0977.1 when co-expressed *in trans* (from a site distant from the vector MCS), we used an inverse PCR approach to construct different plasmids on the basis of pDIA6335. RCd11 (-158 to +386 relative to the first TSS), RCd12 (-158 to +386 relative to the TSS) and RCd10 (-167 to +282 relative to the TSS) with their respective promoter were amplified by PCR and cloned into pDIA6335 linearized by inverse PCR at a site distant from the vector MCS, yielding pDIA6791, pDIA6792 and pDIA6793, respectively.

For subcellular localization of toxin CD0977.1, we used an inverse PCR approach to construct *CD0977.1*-HA-expressing plasmids (pDIA6622) on the basis of pDIA6335 with primers designed to introduce the hemagglutinin HA-tag sequence at the C-terminal part of toxin coding regions, directly upstream of the stop codon (Table S3).

For expression of the CD0912 excisionase, the *CD0912* gene (-37 to + 333 relative to the translational start site) was amplified by PCR and cloned into the linearized pDIA6103 vector, yielding pDIA6867.

For construction of promoter*::phoZ* constructs, the P_1 promoter of RCd11 together with the associated Cdi1_4 riboswitch, the P_2 promoter of RCd11, both the P_1 promoter of RCd11 together with the associated Cdi1_4 riboswitch and the P_2 promoter or both the P_1 promoter of RCd11 together with the associated Cdi1_4 riboswitch and the disrupted P_2 promoter were amplified by PCR and cloned into the linearized pMC358 vector, yielding p097, p098, p099 and p100.

Supplementary references

1. Heap JT, Pennington OJ, Cartman ST, Minton NP. A modular system for Clostridium shuttle plasmids. *J Microbiol Methods* **78**, 79-85 (2009).

2. Soutourina OA*, et al.* Genome-wide identification of regulatory RNAs in the human pathogen *Clostridium difficile*. *PLoS genetics* **9**, e1003493 (2013).
