# Supplementary material for: Type I toxin-antitoxin systems contribute to the maintenance of mobile genetic elements in Clostridioides difficile
Source: Commun Biol. 2020 Nov 27;3:718. doi: 10.1038/s42003-020-01448-5 (PMC7699646; doi:10.1038/s42003-020-01448-5)
Supplement: Supplementary file 3 — Descriptions of Additional Supplementary Files [file 42003_2020_1448_MOESM3_ESM.pdf]

Description of Additional Supplementary Files

Supplementary Data 1

Description: Strains and plasmids used in this study.

Supplementary Data 2

Description: Oligonucleotides used in this study. Restriction sites are underlined. Lowercase bases indicate overlapping sequences. The promoter sequence for T7 polymerase is indicated in italics.

Supplementary Data 3

Description: Source data for graphs in main Figures. The data used for Figures 3a, 3b, 3c, 4, 5b and 5c are included.
